# How does the consecutive use of intraoral scanners affect musculoskeletal health? A preliminary clinical study

**DOI:** 10.1186/s40001-024-01895-4

**Published:** 2024-06-15

**Authors:** KeunBaDa Son, Ji-Min Lee, Jin-Wook Kim, Myoung-Uk Jin, Kyu-Bok Lee

**Affiliations:** 1 Advanced Dental Device Development Institute, Department of Dental Science, Graduate School, Kyungpook National University, Daegu, 41940 Republic of Korea; 2 Department of Oral & Maxillofacial Surgery, School of Dentistry, Kyungpook National University, Daegu, Republic of Korea; 3 Department of Conservative Dentistry, School of Dentistry, Kyungpook National University, Daegu, Republic of Korea; 4 Department of Prosthodontics, School of Dentistry, Kyungpook National University, Daegu, 41940 Republic of Korea

**Keywords:** Intraoral scanner, Musculoskeletal health, Surface electromyography, Muscle fatigue

## Abstract

**Background:**

Minimizing muscle strain and reducing the risk of musculoskeletal disorders associated with intraoral scanner (IOS) usage require ergonomic awareness, device selection, and workplace adjustments in dental practice. This preliminary clinical study aimed to simulate intraoral scanning tasks using wired and wireless IOSs and assess muscle activation and fatigue for both types.

**Materials and methods:**

Fourteen participants performed intraoral scanning tasks using wired and wireless IOSs (i700; MEDIT), with weights of 280 g and 328 g, respectively. The same computer system and software conditions were maintained for both groups (*N* = 14 per IOS group). Electrodes were placed on arm, neck, and shoulder muscles, and maximal voluntary contraction (MVC) was measured. Surface electromyography (EMG) was performed during the simulation, and EMG values were normalized using MVC. The root mean square EMG (%MVC) and muscle fatigue (%) values were calculated. Statistical comparisons were performed using the Mann–Whitney *U* and Friedman tests, with the Bonferroni adjustment for multiple comparisons (*α* = 0.05).

**Results:**

Arm (flexor digitorum superficialis) and neck muscles (left sternocleidomastoid and left splenius capitis) showed significantly higher EMG values with wireless IOS (*P* < 0.05). The neck (left sternocleidomastoid and right levator scapulae) and shoulder muscles (right trapezius descendens) demonstrated significantly higher muscle fatigue with wireless IOS (*P* < 0.05).

**Conclusions:**

The consecutive use of heavier wireless IOS may increase the risk of muscle activation and fatigue in certain muscles, which may have clinical implications for dentists in terms of ergonomics and musculoskeletal health.

## Introduction

In recent years, the development of intraoral scanners (IOSs) has brought about significant changes in the field of dentistry, revolutionizing the impression acquisition process [1, 2]. With its accurate and efficient digital scanning of the oral cavity, intraoral scanning technology has replaced conventional impression materials and techniques [3]. The benefits of this technology include reduced patient discomfort, improved accuracy, and shortened processing times [4, 5].

Despite these advantages, the repetitive movements required during intraoral scanning can lead to muscle tension and fatigue, resulting in discomfort and pain in the neck, shoulder, and arms [6]. This is further compounded by the consecutive use of various medical devices for treatment, such as handpieces and ultrasonic scalers, which also require repetitive and sustained use of the arm, neck, and shoulder muscles [7, 8]. These movements can cause muscle tension and fatigue, leading to discomfort and pain [9–12]. Therefore, it is essential to evaluate the activation and fatigue levels of the arm, neck, and shoulder muscles associated with the consecutive use of various medical devices, including IOSs, to promote the safety of dentists and provide high-quality patient care [13].

A previous study has demonstrated that intraoral scanning can also present ergonomic challenges and musculoskeletal risks for dentists [6]. These risks arise due to the need for dentists to hold dental devices in specific positions for extended periods, which can lead to increased muscle activation and fatigue [14]. Another study investigated muscle contraction and fatigue levels associated with various tasks related to patient treatment, such as tooth preparation tasks [15]. The researchers warned about the risk of musculoskeletal disorders linked to dentists' posture and sustained performance [15]. To address this issue, it is essential to evaluate the musculoskeletal risks associated with consecutive use of IOSs, specifically focusing on the activation and fatigue levels of the arm [flexor digitorum superficialis (FDS) and extensor digitorum communis (EDC)], neck [sternocleidomastoid muscle (SCM) and splenius capitis (SC)], and shoulder [trapezius descendens (T)] muscles [16–19]. Dental work during treatment involves various muscles, including those in the arms, neck, shoulders, and back. The arm muscles, FDS and EDC, are used when flexing the wrist and applying force to the grip while utilizing dental instruments [16]. The SCM, a neck muscle, is engaged when turning the head, while the SC is activated when bending the head to observe the patient’s mouth. Both muscles are essential for maintaining proper posture during dental procedures. As for the shoulder muscles, the T is utilized to elevate the shoulder for supporting heavy medical devices carried over from the arm and has frequently been employed by dentists in surface electromyography (EMG) measurements to assess musculoskeletal health [16–19].

The predominant approach to assessing musculoskeletal health involves analyzing EMG signals during tasks and normalizing these against the maximal voluntary contraction (MVC) of specific muscles [20, 21]. The techniques for measuring and calculating MVC are essential [22]. Typically, MVC is calculated by having participants perform actions that provide direct resistance to maximally contract the muscle, while measuring the resultant MVC signal [23]. For individuals experiencing musculoskeletal pain, measuring sub-MVC without direct resistance has demonstrated high reliability [24, 25]. Variations in MVC measurements, influenced by differing postures and tools, can lead to discrepancies in EMG readings [26]. Various methods and conditions for maximal muscle contraction exist in MVC calculations, underscoring the importance of condition control in experiments [27]. An alternative MVC determination method relies on specific factors, calculated without direct participant measurement [28]. This approach utilizes video observation to determine factors such as body weight, muscle cross-sectional area, and limb length [29]. Advanced computer models, integrating EMG and motion capture data, facilitate musculoskeletal modeling and MVC estimation [30]. Therefore, selecting appropriate MVC measurement methods to normalize EMG signals, considering experimental conditions, is vital for minimizing errors.

Wired and wireless IOSs have emerged in the market [31–34]. IOSs are extensively used in various dental applications, including the fabrication of dental prosthetics, the creation of dental casts for orthodontic purposes, and the generation of guide templates for implant surgery planning [31]. Their use is increasingly becoming a norm in dental clinical practices [33, 34]. While wired IOSs necessitate a direct connection to a computer system, wireless IOSs operate via Bluetooth technology, removing the need for physical connections. Although wireless IOSs provide enhanced mobility and flexibility, they are generally heavier and bulkier than their wired counterparts, potentially increasing the risks of muscle tension and fatigue. Assessing muscle activation and fatigue during various dental procedures is a crucial factor in evaluating dentists’ musculoskeletal health [35–40]. Despite the widespread adoption of intraoral scanning technology, little is known about the differences in muscle activation and fatigue associated with using distinct types of scanners. Consequently, it is vital to evaluate the ergonomic and musculoskeletal risks linked to the utilization of wired and wireless IOSs. Comprehending the disparities in muscle activation and fatigue related to both types of IOSs can contribute to the development of ergonomic guidelines and recommendations for dentists, thereby promoting their safety while delivering high-quality patient care. According to prior studies [5, 40], IOS weight ranges from 113 to 585 g, indicating that dentists need to exercise caution when using IOS consecutively in clinical practice. This variation suggests that some IOS models may be heavier than many traditional dental tools, such as explorers, probing tools, handpieces for tooth preparation, and handpieces used in implant placement. Consequently, dentists should be cautious when using IOSs consecutively in clinical settings, considering the potential ergonomic impact of their weight on musculoskeletal health.

Despite their importance, the effects of the consecutive use of wired and wireless IOSs on the musculoskeletal system remain unexplored. Therefore, this preliminary clinical study aimed to evaluate muscle activation and fatigue associated with wired and wireless IOSs during task simulations related to intraoral scanning. The null hypothesis of this study was that the consecutive use of wired and wireless IOSs does not affect muscle contraction and fatigue levels during intraoral scanning-related tasks.

## Methods

### Selection of participants

This clinical trial was approved by the Clinical Trial Ethics Committee of Kyungpook National University Dental Hospital (IRB No. KNUDH-2021-04-04-00). The study was conducted in compliance with the 1964 Helsinki Declaration and its subsequent amendments. Before inclusion, all participants provided informed written consent.

The sample size was determined using power software (G*Power version 3.1.9.2; Heinrich-Heine-Universität Düsseldorf), with 14 individuals selected for each IOS group based on a previous pilot study with 3 participants who utilized the same method (actual power = 99.8%; power = 99%; *α* = 0.05). Recruitment for the clinical trial commenced in June 2021, targeting clinicians who graduated from the School of Dentistry and had experience in creating dental prosthetics using digital dental workflows. The clinical trial began in October 2021, with all evaluations being carried out within the School of Dentistry. The trial was successfully completed in November 2022.

The participants recruited were right-handed male and female dentists with experience in digital dentistry, specifically in producing dental restorations using IOS and digital dental workflows. The study included eight men and six women with a mean age of 29.7 ± 4.1 years, height of 169.1 ± 5.5 cm, weight of 67.2 ± 10.1 kg, and clinical dental experience of 3.0 ± 1.5 years. The detailed physiological data are presented in Table 1. The experimental order was randomly assigned to each participant. Participants were randomly allocated using the program (Random Allocation; Isfahan University of Medical Sciences, Isfahan, Iran).

**Table 1:** Physiological parameters

Parameters	All participants
Gender	Male: 8, female: 6
Age (years)	29.7 ± 4.1
Height (cm)	169.1 ± 5.5
Weight (kg)	67.2 ± 10.1
Dental clinical experience in prosthodontics (years)	3.0 ± 1.5
IOS operating experience (years)	1.14 ± 0.8
IOS usage frequency in last month (patient application)	3.5 ± 5.2 times

Data are presented as mean ± standard deviation

IOS; intraoral scanner

All participants had no history of musculoskeletal disorders related to the musculoskeletal system. Comprehensive interviews focusing on musculoskeletal disorders were conducted with the participants. The questions included: Have you experienced any musculoskeletal pain or fatigue during recent dental clinical activities? If there was fatigue, did it persist beyond nocturnal rest? Participants who reported persistent musculoskeletal discomfort even after rest were excluded from the study. The questionnaire was meticulously designed to identify symptoms related to musculoskeletal issues commonly exacerbated by intraoral scanner use, focusing on the neck, shoulders, arms, and back. This exclusion criterion aimed to minimize confounding variables and ensure that observed effects on muscle activation and fatigue were directly attributable to IOS usage.

To minimize the potential impact of muscle thickness, which may vary by gender, participants were selected to ensure similarity in height and age. Although the present study involved the same participants using two different types of IOS, the relative effect of muscle thickness was deemed less significant. To counter any potential influence of muscle morphology or thickness on cumulative fatigue, each session included a 10-min rest period, and sessions using different scanners were conducted after a full day's rest. Direct fatigue levels were monitored through interviews, with experiments proceeding only if participants reported no cumulative muscle fatigue.

### Muscle activity monitoring

**Fig. 1:**
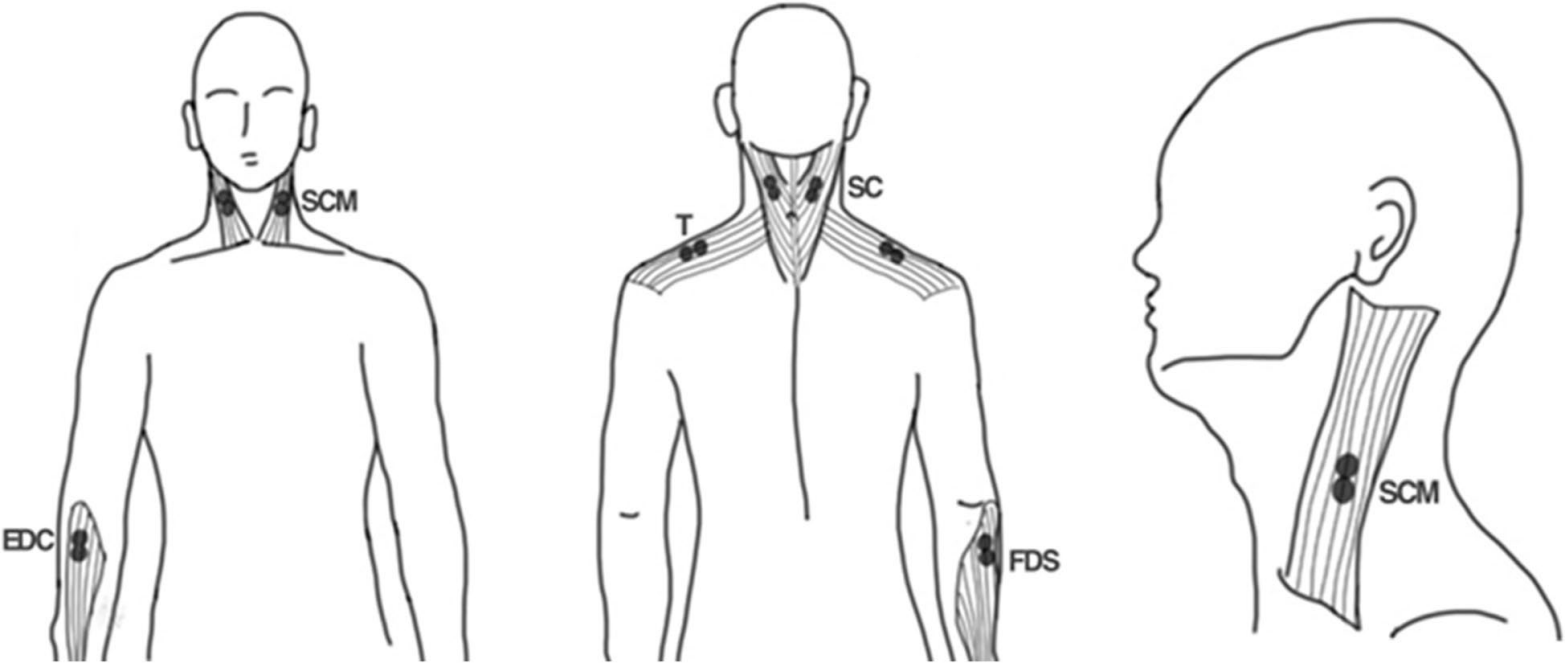
Schematic representation of electrode attachment positions. EDC, extensor digitorum communis; FDS, flexor digitorum superficialis; SCM, sternocleidomastoid muscle; SC, splenius capitis; T, trapezius descendens

To monitor muscle activity during dental procedures, the placement of electrodes used in previous studies was referenced [6–10, 13–16]. Surface EMG measurements were performed on various muscles, including the arm (EDC and FDS), neck (SCM and SC), and shoulder muscles (T) (Fig. 1). Each muscle was measured using a pair of 20-mm diameter electrodes attached to the surface of the skin using a pre-gelled adhesive (Covidien, Mansfield, USA). Before the electrodes were applied, the skin was cleaned using a 70% isopropyl alcohol swab to ensure proper adhesion. The electrodes were placed over the muscle fibers according to the Surface Electromyography for the Non-Invasive Assessment of Muscles (SENIAM) protocol, with a 20-mm distance between two electrodes [6–10]. The grounding electrode was attached to the coracoid process of the left scapula (Fig. 1) [6–10].

In this study, four specific muscles were selected based on previous research investigating the ergonomics of dentists during intraoral dental work [16–19]. The EDC and FDS muscles in the arm, responsible for finger and hand extension, respectively, were chosen due to their frequent use during intraoral scanning tasks. The SCM and SC muscles in the neck were selected because they are commonly employed to maintain head and neck posture during dental procedures. The T is utilized to elevate the shoulder, supporting heavy medical devices carried over from the arm. The EDC electrode was positioned between the lateral epicondyle of the humerus and the styloid process of the ulna, while the FDS electrode was placed at the midpoint of the medial border of the coronoid process of the ulna and the medial epicondyle of the humerus (Fig. 1) [11, 12]. The SCM electrode was attached to the sternal portion of the muscle, at one-third of the distance from the mastoid process to the sternal notch (Fig. 1) [11]. The SC electrode was placed at the midpoint between the mastoid process and the seventh cervical vertebra, and the T muscle electrode was positioned at the midpoint between the acromion and the seventh cervical vertebra (Fig. 1) [11]. These specific muscle groups and electrode placements were chosen to accurately assess the muscle activation and fatigue levels during intraoral scanning procedures, providing valuable insights into the ergonomic risks associated with the use of wired and wireless IOSs.

The electrodes were connected to an EMG measurement system (WEMG-8; LAXTHA). The signal from each channel was amplified to 244 μV through the EMG preamplifier in the measurement system and digitized at a sampling rate of 1000 Hz using an AD converter.

### Maximal voluntary contraction measurements

A common approach to normalize EMG data is to use MVC measurements. MVC measurements were conducted according to the guidelines established by the SENIAM Protocol [12, 15] and were performed with participants seated on a chair that provided back support. For measurements involving the arm muscles, the participant’s forearm was placed on a desk, and the elbow was flexed at a 90° angle. To measure the EDC muscle in the arm, maximal resistance was applied during hand and finger extension, whereas the FDS muscle was measured using a grip strength meter with maximal force applied to the fingers and palms. The SCM muscle in the neck was measured by providing maximal resistance during head rotation to the left and right with both arms lowered, whereas the SC muscle was measured by providing maximal resistance when tilting the head down and then lifting it up. The T muscle in the shoulder was measured by providing maximal resistance while lifting the shoulder. Three trials were conducted for each muscle, with a 5-s interval between each trial. The MVC value was then determined by selecting the highest value, which was used to normalize the EMG activity.

**Fig. 2:**
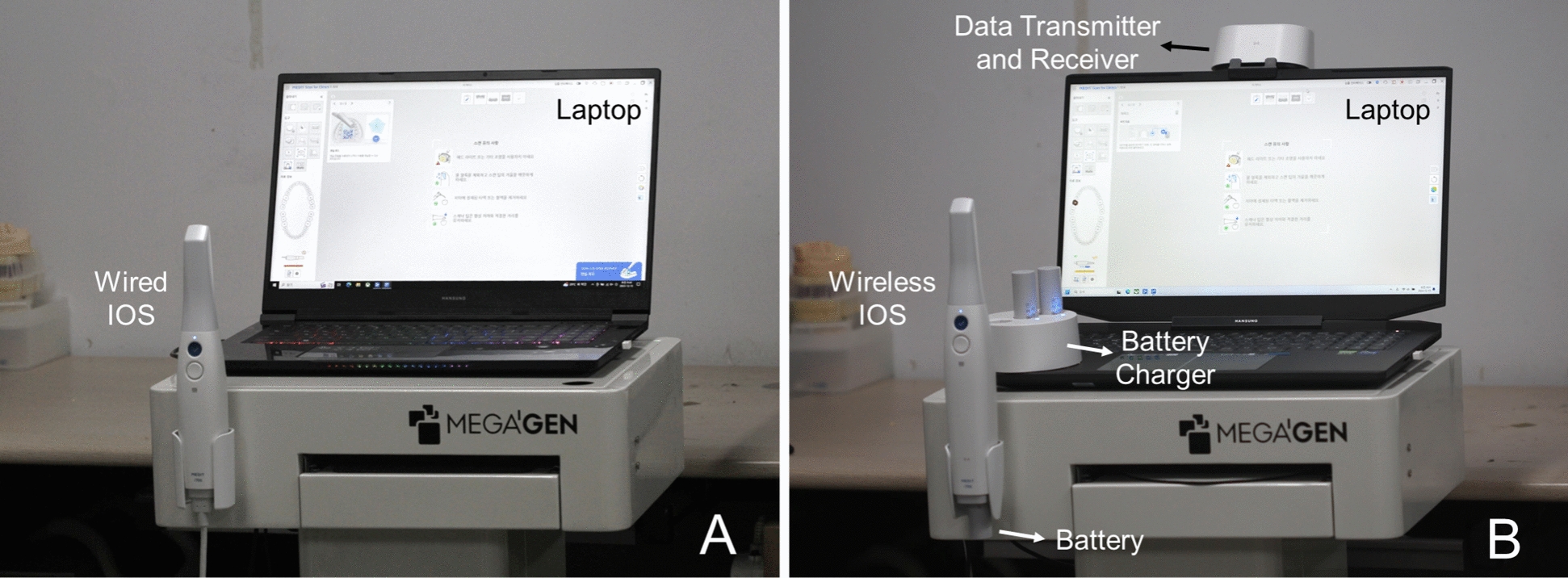
Intraoral scanners (IOS) used in this study. **A** Wired IOS. **B** Wireless IOS

**Fig. 3:**
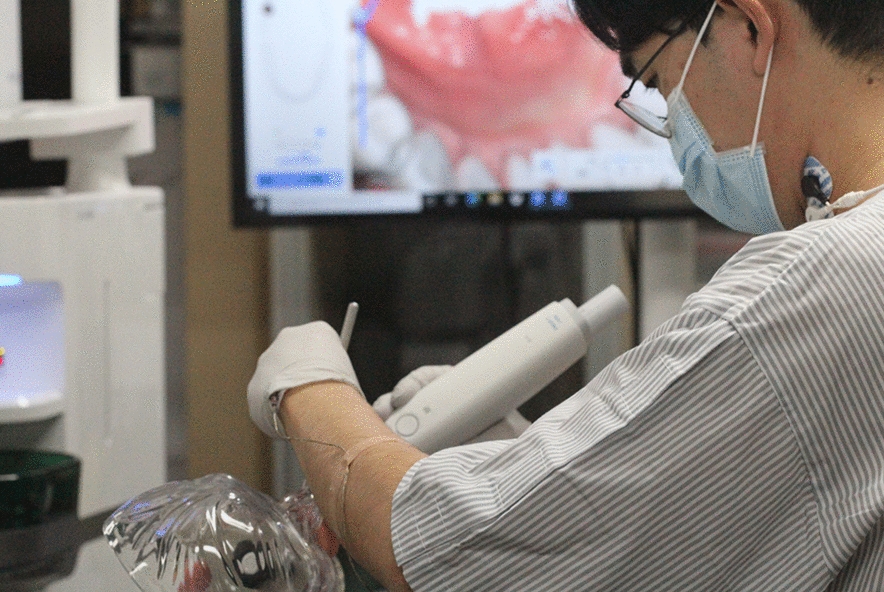
Scanning simulation using a wireless intraoral scanner

After the MVC measurement, participants performed intraoral scanning simulations on a dental manikin and typodont (Simple Manikin III, NISSIN) installed in a dental unit chair system (MEGAGEN). Muscle activation was recorded using eight EMG channels. Wireless and wired IOS (i700; MEDIT) were utilized for intraoral scanning simulations (Fig. 2). According to the manufacturer, the wireless type is an IOS with a module added for wireless data transmission to the same optical system as the wired type, and there is no difference in appearance, such as the size of the scan tip, other than the weight difference (328 g for wireless IOS and 280 g for wired IOS). A computer system with specifications superior to those recommended by the manufacturer was employed, as computer specifications can significantly impact scanning speed. All experiments with wireless and wired IOS (i700; MEDIT) were conducted using the same software version (MEDIT) on the same computer. Participants adjusted the patient chair and dental stool to ensure comfort during the intraoral scanning process. The wireless IOS was fully charged prior to use, with the scanning procedure displayed on a chairside monitor of dental unit chair system connected to a laptop (Fig. 3). In contrast, the wired IOS, due to its cord constraints, projected the scanning process directly onto the laptop (Fig. 2).

### Intraoral scanning simulations

A total of 14 participants received approximately 30 min of education on the operation of the two types of IOSs and performed one practice session per IOS (Fig. 2). The experimental order was randomly assigned to each participant, and the next type of IOS experiment was conducted after a rest day. Furthermore, the health status of each participant was assessed, and through interviews, experiments were carried out when participants were in their optimal condition. Recognizing the potential impact of intraoral scanning proficiency on muscle fatigue, this study selectively recruited participants with approximately 3 years of general dental clinical experience and a minimum of one year of specialized experience in intraoral scanning. Additionally, the duration of work was strictly limited to no more than 10 min per session. This constraint was rigorously monitored and enforced by the investigators to ensure compliance and participant awareness.

Wireless and wired IOSs were performed for four repeated tasks per participant (Fig. 3). The decision to limit the number of operations to four per IOS was based on findings from a pilot study aimed at determining adequate sample sizes. It was observed that conducting more than four tasks in a single day resulted in fatigue that could not be alleviated even with breaks longer than 10 min. Consequently, it was established that more than four tasks per session would compromise the validity of the results due to excessive fatigue accumulation. To prevent cumulative muscle fatigue in each session, a break of at least 10 min was taken before moving on to the next session, which was conducted only when the participant did not feel fatigued. The scanning process began by scanning the maxilla of the typodont model prior to scanning the mandible. The scan strategy was to scan from the left maxillary second molar to the right maxillary second molar in the occlusal and cross-sectional directions, then sequentially scan in the contralateral direction and complete the maxillary scan in the ipsilateral direction. The complete mandibular arch was scanned using the same scan strategy as that used for the maxillary arch. The participants consecutively checked for defects in the scanned area during the scanning process and completed the complete arch scan to ensure that there were no holes in the tooth area. An investigator monitored the participants’ scanning process in real-time and observed the inappropriate use of the IOSs. Additionally, one investigator (K.S.) recorded muscle activation in real-time only when the participant performed an action for simulation and did not record muscle activation otherwise. Working time for both wired and wireless IOSs was meticulously recorded, focusing exclusively on the periods when participants actively engaged with the IOS, in alignment with muscle activation recording. The working time was noted as the duration required to complete the scan in each of the four repetitions.

### Evaluation of muscle activation and fatigue

Muscle activation and fatigue were evaluated using an EMG measurement software (EMG-Works 4.0; Delsys Inc) during the simulated dental tasks. EMG data were normalized and expressed as a percentage of each muscle’s MVC using the following formula [6–10, 15]:

$$RMS\, EMG(\%MVC) = \frac{Muscle\, activation\, during\, tasks \left(\mu \text{V}\right)}{MVC}\times 100.$$

The root mean square (RMS) EMG (%MVC) represents the level of muscle activation during tasks relative to MVC. A higher RMS EMG (%MVC) value may indicate an increased risk of musculoskeletal disorders. Previous research classified ergonomic risk level based on the level of activation of each muscle: 0–10% MVC indicates “low risk”, 11–20% indicates “medium risk”, and 21% or higher indicates “high risk” [6–8, 10, 15].

Muscle fatigue was evaluated by analyzing the median frequency (MF) of the EMG signal, with a decrease in MF indicating an increase in muscle fatigue [10–12]. The MF was obtained by applying a fast Fourier transform to the EMG signal and calculating it within a frequency range of 20–500 Hz. Muscle fatigue was calculated by comparing the MF in the second half of the total work time to the MF in the first 60 s using the following formula [6–10, 15]:

$$Muscle\, fatigue\left(\text{\%}\right) = \frac{MEF\, in\, the\, second\, 60\, seconds\, - MEF\, in\, the\, first\, 60\, seconds}{MEF\, in\, the\, first\, 60\, seconds}\times 100.$$

If the MF in the second half of 60 s is lower than that in the first 60 s, resulting in a negative value, muscle fatigue is considered to have increased [6–11]. In this study, to analyze EMG activity across consecutive tasks, the mean graph of the median frequency of EMG signals was derived for a 60-s interval at the midpoint of each task's inception and conclusion.

### Statistical analysis

All data were analyzed using statistical analysis software (Statistical Package for the Social Sciences ver. 25.0; IBM) with a significance level of *α* = 0.05. First, the normality of the data was examined using the Shapiro–Wilk test, which indicated that the data did not follow a normal distribution. The Wilcoxon rank-sum test was used to compare the wired and wireless IOSs in terms of EMG and muscle fatigue. To compare the differences in EMG and muscle fatigue during repeated intraoral scanning simulations in the fourth session, the Friedman test was used with a significance level of *α* = 0.05. The Bonferroni correction was applied for multiple comparisons, with a significance level of *α* = 0.05.

To determine the influence of consecutive usage and working duration on muscle activation and fatigue, a correlation analysis was conducted (*α* = 0.05). Spearman’s rank correlation coefficient was used for this analysis. Correlation strength was categorized as follows: a coefficient of ± 0.3 or lower signified a slight correlation, ± 0.3 to 0.5 indicated a low correlation, ± 0.5 to 0.7 suggested a moderate correlation, ± 0.7 to 0.9 represented a high correlation, and a coefficient of ± 0.9 or higher denoted a very high correlation [41]. Multivariate variance tests with partial eta-squared from the Kruskal–Wallis and Mann–Whitney *U* tests were performed to determine the effects of independent variables and interactions.

## Results

There was no statistically significant difference in the working time during the fourth iteration of the two IOSs (Table 2; *p* > 0.05). The consecutive use of IOSs did not demonstrate a significant correlation with RMS EMG and muscle fatigue (Table 3; *p* > 0.05). However, each session’s working time showed a slight but significant positive correlation with the RMS EMG of the right SCM muscle (Table 3; *p* = 0.023; correlation coefficient = 0.215). Furthermore, the working time in each session correlated positively and significantly with muscle fatigue in the right SCM (Table 3; *p* = 0.005; correlation coefficient = 0.264) and left SC (Table 3; *p* = 0.042; correlation coefficient = 0.193).

**Table 2:** Comparison of working time (seconds) for wireless and wired intraoral scanners in the first, second, third, and fourth iterations

Trial no	Intraoral scanner type	Mean	SD	Median	95% confidence interval		p*
					Lower limit	Upper limit	
1	Wireless	535.5	91.2	482.8	588.1	535.5	0.888
	Wired	530.3	99.1	473.0	587.6	530.3	
2	Wireless	435.0	73.8	392.4	477.7	435.0	0.525
	Wired	418.2	63.7	381.4	455.0	418.2	
3	Wireless	379.0	77.5	334.3	423.8	379.0	0.900
	Wired	382.3	57.9	348.8	415.8	382.3	
4	Wireless	352.0	56.8	319.2	384.9	352.0	0.453
	Wired	335.6	57.1	302.6	368.6	335.6	

^*^Significant difference between two intraoral scanners determined by the Wilcoxon rank-sum test, *p* < 0.05

**Table 3:** Correlation analysis between factors of consecutive usage and working time of intraoral scanners and muscle activation and fatigue

Muscle activation and fatigue	Muscle type	Consecutive use		Working time (s)	
		Correlation coefficient	*P*	Correlation coefficient	*P*
RMS EMG (%MVC)	EDC	0.067	0.482	0.117	0.220
	FDS	0.034	0.719	0.108	0.256
	Left SCM	0.013	0.895	0.037	0.700
	Right SCM	0.024	0.802	0.215	0.023*
	Left SC	0.024	0.800	0.003	0.976
	Right SC	0.038	0.692	0.125	0.190
	Left T	0.039	0.681	0.001	0.994
	Right T	0.105	0.272	0.097	0.311
Muscle fatigue (%)	EDC	0.028	0.770	0.027	0.781
	FDS	0.01	0.916	0.100	0.292
	Left SCM	0.088	0.354	0.065	0.497
	Right SCM	0.067	0.482	0.264	0.005*
	Left SC	0.003	0.977	0.193	0.042*
	Right SC	0.062	0.513	0.084	0.378
	Left T	0.042	0.660	0.090	0.347
	Right T	0.062	0.519	0.074	0.440

EDC, extensor digitorum communis; FDS, flexor digitorum superficialis; SCM, sternocleidomastoid muscle; SC, splenius capitis; T, trapezius descendens

^*^Significant correlations determined using Spearman's rank correlation coefficient analysis (*p* < 0.05)

In both wired and wireless IOSs, the RMS EMG values did not increase significantly during the fourth iteration. However, muscle fatigue showed a significant increase in specific muscles (Tables 4, 5, and 6). Significant increases in muscle fatigue were observed in both arm [FDS in both wired (*p* = 0.016) and wireless (*p* = 0.008) IOSs and EDC in wired IOS (*p* = 0.014)] and shoulder muscles (right T in both wired (*p* = 0.002) and wireless (*P* = 0.017) IOSs).

**Table 4:** Comparison of muscle activation and fatigue of arm muscles during consecutive use of wired and wireless intraoral scanners

Arm muscle type	Intraoral scanner type	Number of iterations	RMS EMG (%MVC)			p**	Muscle fatigue (%)			p**
			Median	95% confidence interval			Median	95% confidence interval		
				Lower	Upper			Lower	Upper	
Extensor digitorum communis	Wire type	1	11.65	10.22	13.73	0.091	2.13^A^	− 3.82	13.96	0.362
		2	12.67	10.61	14.09		− 3.56^AB^	− 8.79	− 1.77	
		3	13.16	11.24	14.57		− 6.76^AB^	− 9.59	0.21	
		4	12.30	10.99	15.06		− 9.29^B^	− 12.80	− 5.34	
		P*	0.733				0.016			
	Wireless type	1	14.04	11.64	16.46		− 2.84^A^	− 8.29	4.48	
		2	13.94	10.97	16.38		− 2.17^A^	− 7.38	1.51	
		3	13.60	11.29	16.58		− 7.02^AB^	− 9.62	− 4.94	
		4	14.15	11.53	15.61		− 11.09^BC^	− 16.90	− 7.80	
		P*	0.962				0.008			
Flexor digitorum superficialis	Wire type	1	7.27	5.92	9.55	0.008 (Wire < Wireless)	− 1.95^A^	− 5.36	9.83	0.359
		2	7.07	6.20	11.51		− 4.58^AB^	− 8.97	− 2.70	
		3	8.11	6.64	12.32		− 7.87^AB^	− 14.69	− 5.30	
		4	7.32	6.32	11.30		− 9.70^B^	− 16.04	− 6.24	
		P*	0.842				0.014			
	Wireless type	1	10.81	8.31	14.86		− 2.05	− 11.48	3.13	
		2	10.73	7.89	15.12		− 6.38	− 13.51	− 3.66	
		3	11.76	8.2	16.36		− 9.39	− 20.50	− 3.96	
		4	10.83	8.15	15.19		− 11.86	− 15.23	− 7.53	
		P*	0.978				0.095			

EMG, electromyography; MVC, maximal voluntary contraction; RMS, root mean square

^*^Statistical significance of the EMG and muscle fatigue increase during consecutive intraoral scanning in the fourth session determined using the Friedman test, *p* < 0.05. Significant differences among the fourth session are indicated by different capital letters, determined using Bonferroni correction, *p* < 0.05

^**^Significance determined using Wilcoxon rank-sum test for comparison of wired and wireless intraoral scanners in EMG and muscle fatigue, *p* < 0.05

**Table 5:** Comparison of muscle activation and fatigue of neck muscles during consecutive use of wired and wireless intraoral scanners

Neck muscle type	Intraoral scanner type	Number of iterations	RMS EMG (%MVC)			p**	Muscle fatigue (%)			p**
			Median	95% confidence interval			Median	95% confidence interval		
				Lower	Upper			Lower	Upper	
Left sternocleidomastoid muscle	Wire type	1	6.43	5.01	9.04	0.015 (Wire < Wireless)	− 0.66	− 9.19	6.79	< 0.001 (Wire < Wireless)
		2	5.87	5.06	9.30		− 1.85	− 17.42	6.12	
		3	5.74	5.11	9.42		− 0.92	− 9.65	3.32	
		4	5.43	5.18	8.01		− 1.21	− 8.65	4.51	
		P*	0.99				0.789			
	Wireless type	1	7.15	6.25	11.17		− 11.60	− 21.94	3.14	
		2	7.22	6.07	11.55		− 12.56	− 16.61	− 3.02	
		3	7.96	6.35	12.39		− 13.64	− 28.77	− 11.55	
		4	7.88	6.55	12.23		− 11.44	− 24.54	− 7.02	
		P*	0.982				0.578			
Right sternocleidomastoid muscle	Wire type	1	8.10	5.86	10.22	0.192	5.70	− 6.39	17.48	0.871
		2	7.19	5.58	10.01		0.46	− 6.17	6.31	
		3	7.55	5.70	10.35		0.68	− 8.16	18.57	
		4	7.85	5.51	10.17		0.97	− 8.39	5.74	
		P*	0.99				0.508			
	Wireless type	1	8.83	6.26	11.81		4.71	− 0.56	8.49	
		2	9.88	6.52	12.08		0.11	− 1.84	6.77	
		3	9.45	6.15	11.85		− 0.13	− 3.70	6.94	
		4	9.42	6.54	12.36		− 0.73	− 5.20	4.01	
		P*	0.991				0.429			
Left splenius capitis	Wire type	1	9.18	6.91	11.65	< 0.001 (Wire < Wireless)	4.42	− 2.21	11.57	0.124
		2	9.17	7.12	12.91		4.41	− 4.47	15.03	
		3	10.02	7.51	13.47		8.96	3.28	27.06	
		4	8.14	6.04	15.74		11.77	2.60	18.55	
		P*	0.868				0.475			
	Wireless type	1	13.14	10.65	18.12		2.17	0.01	21.04	
		2	13.86	10.92	17.92		0.25	− 3.68	15.80	
		3	11.16	9.46	18.08		0.50	− 3.33	9.86	
		4	11.74	10.08	16.00		− 0.43	− 2.58	10.62	
		P*	0.936				0.479			
Right splenius capitis	Wire type	1	9.90	5.48	19.58	0.124	− 1.24	− 8.41	7.44	0.043 (Wire < Wireless)
		2	7.93	6.32	12.29		− 4.13	− 11.29	− 1.23	
		3	8.29	5.36	18.45		− 6.39	− 13.22	− 2.76	
		4	8.42	5.47	20.47		− 8.13	− 13.84	− 1.95	
		P*	0.949				0.419			
	Wireless type	1	11.14	8.04	15.27		− 6.80	− 13.70	3.21	
		2	10.59	7.79	14.92		− 8.06	− 14.04	− 0.22	
		3	9.85	7.64	17.85		− 10.32	− 27.56	1.39	
		4	11.85	8.40	16.12		− 14.63	− 20.99	− 8.75	
		P*	0.98				0.291			

EMG, electromyography; MVC, maximal voluntary contraction; RMS, root mean square

^*^Statistical significance of the EMG and muscle fatigue increase during consecutive intraoral scanning in the fourth session determined using the Friedman test, *p* < 0.05. Significant differences among the fourth session are indicated by different capital letters, determined using Bonferroni correction, *p* < 0.05

^**^Significance determined using Wilcoxon rank-sum test for comparison of wired and wireless intraoral scanners in EMG and muscle fatigue, *p* < 0.05

**Table 6:** Comparison of muscle activation and fatigue of shoulder muscles during consecutive use of wired and wireless intraoral scanners

Shoulder muscle type	Intraoral scanner type	Number of iterations	RMS EMG (%MVC)			p**	Muscle fatigue (%)			p**
			Median	95% confidence interval			Median	95% confidence interval		
				Lower	Upper			Lower	Upper	
Left trapezius descendens	Wire type	1	10.65	8.33	16.42	0.178	2.42	− 3.67	17.12	0.084
		2	10.91	8.48	15.58		− 0.23	− 3.37	8.19	
		3	11.49	9.40	17.23		− 1.27	− 8.13	8.06	
		4	11.45	9.16	17.66		− 2.32	− 15.73	3.91	
		P*	0.865				0.318			
	Wireless type	1	11.79	9.97	18.04		− 0.29	− 4.51	7.01	
		2	11.28	9.55	17.18		− 0.48	− 6.02	7.80	
		3	12.88	11.14	17.90		− 1.19	− 6.80	3.74	
		4	13.03	10.64	19.29		− 3.44	− 8.52	6.30	
		P*	0.93				0.277			
Right trapezius descendens	Wire type	1	14.47	12.44	18.10	0.362	− 0.71^A^	− 3.61	3.90	0.009 (Wire < Wireless)
		2	14.81	12.52	18.39		− 2.91^AB^	− 7.21	− 0.21	
		3	14.31	12.91	19.65		− 5.24^AB^	− 7.49	− 1.12	
		4	15.52	13.66	19.90		− 9.01^B^	− 11.54	− 3.99	
		P*	0.855				0.002			
	Wireless type	1	15.06	12.55	19.98		− 4.36^A^	− 11.20	3.81	
		2	15.73	13.26	20.56		− 4.64^AB^	− 9.05	− 3.08	
		3	14.86	13.42	20.20		− 8.04^AB^	− 11.96	− 5.73	
		4	17.27	14.53	20.91		− 9.56^B^	− 13.92	− 7.78	
		P*	0.816				0.017			

EMG, electromyography; MVC, maximal voluntary contraction; RMS, root mean square

^*^Statistical significance of the EMG and muscle fatigue increase during consecutive intraoral scanning in the fourth session determined using the Friedman test, *p* < 0.05. Significant differences among the fourth session are indicated by different capital letters, determined using Bonferroni correction, *p* < 0.05

^**^Significance determined using Wilcoxon rank-sum test for comparison of wired and wireless intraoral scanners in EMG and muscle fatigue, *p* < 0.05

Based on the results of ergonomic risk level analysis using muscle activation, medium-risk levels were observed in specific muscles (Tables 4–6). The RMS EMG values for the arm (EDC) and shoulder muscles (left and right T) were at a medium-risk level for both wired and wireless IOSs. In addition, the neck muscle (SC) exhibited medium-risk levels only in RMS EMG values for wireless IOSs.

In specific muscles, significantly higher RMS EMG values and muscle fatigue were observed with wireless IOS than with wired IOS (Tables 4–6). The RMS EMG values were significantly higher in the arm (EDC, *p* = 0.008) and neck muscles (left SCM, *p* = 0.015; left SC, *p* < 0.001) with wireless IOS. Muscle fatigue was significantly higher in the neck (left SCM, *p* < 0.001; right SC, *p* = 0.043) and shoulder muscles (right T, *p* = 0.009) with wireless IOS.

**Fig. 4:**
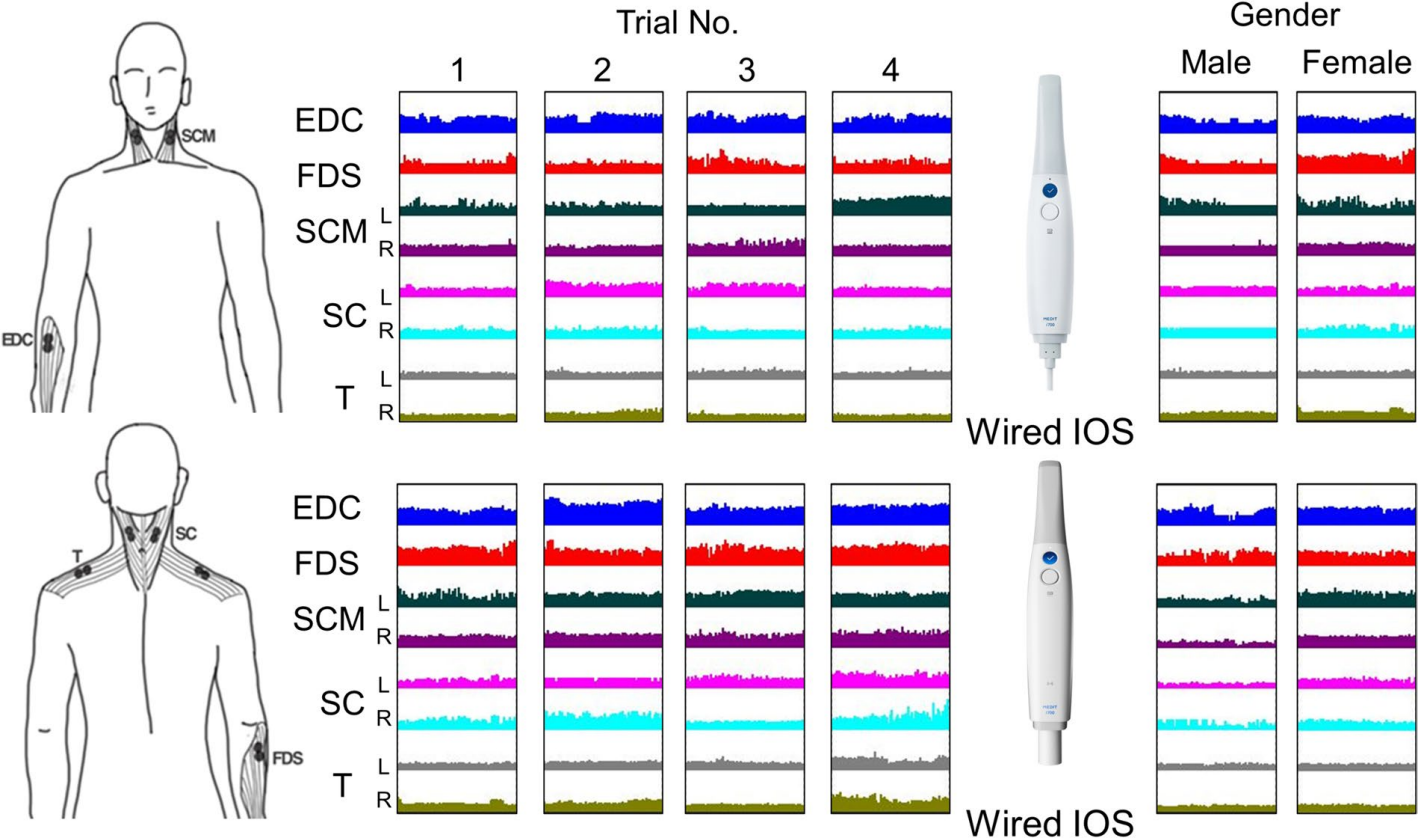
Comparison of the median frequency of EMG signals in muscles during four consecutive tasks using both wired and wireless intraoral scanners (IOSs) in male and female participants. EDC, extensor digitorum communis; FDS, flexor digitorum superficialis; SCM, sternocleidomastoid muscle; SC, splenius capitis; T, trapezius descendens

The mean graph of the median frequency of EMG signals for the 60-s midpoint of the intraoral scanning task facilitates a clear comparison of muscle activity (Fig. 4). When comparing wired and wireless IOSs, increased EMG signals in the arm muscles (EDC and FDS) were discerned in all four consecutive tasks with the wireless IOS. In the shoulder muscles (SC and T), no significant disparity was noted between wired and wireless IOSs in the initial three consecutive tasks; however, the fourth task with the wireless IOS exhibited a pronounced surge in EMG signals in the right SC muscle. In contrast, with the wired IOS, a substantial escalation in EMG signals was detected in the SCM in the fourth repetition. In terms of gender differences in EMG signals, except for the T muscle, higher EMG readings were recorded in female participants.

The results of the multiple-factor analysis showed that both the IOS factor (*p* < 0.001) and the number of iterations factor (*p* < 0.001) had a significant impact on the RMS EMG values and muscle fatigue.

## Discussion

The present study aimed to compare muscle activation and fatigue of right-handed male and female dentists during intraoral scanning simulation using wired and wireless IOSs. The results showed that muscle fatigue increased significantly in specific muscles, including the arm (FDS and EDC) and shoulder muscles (right T), during the fourth iteration of scanning simulations with both wired and wireless IOSs. In addition, the wireless IOS resulted in significantly higher RMS EMG values and muscle fatigue in some specific muscles (EDC, left SCM, left SC, and right T) than those with the wired IOS. Furthermore, multiple-factor analysis showed that both the IOS factor and the number of iterations had a significant impact on RMS EMG values and muscle fatigue. Consequently, the continuous use of both wired and wireless IOSs influenced muscle contraction and fatigue levels during intraoral scanning-related tasks, such that the null hypothesis was rejected (*p* < 0.05) (Tables 4–6). The consecutive use of IOSs did not show a significant correlation with RMS EMG and muscle fatigue (*p* > 0.05) (Table 3). However, each session’s working time displayed a slight but notable positive correlation with both RMS EMG and muscle fatigue in the right SCM and left SC muscles (*p* < 0.05) (Table 3). This suggests that rest intervals between consecutive uses may have played a role in reducing the correlation between RMS EMG and muscle fatigue, even over four sessions. It is posited that the uninterrupted duration of each session until the completion of the assigned task might have led to this specific muscle correlation. These findings imply that reducing the length of individual work sessions and incorporating frequent rest periods can effectively diminish muscle strain. This supports the strategy of optimizing work–rest cycles to mitigate muscle fatigue during extended intraoral scanning activities.

The findings of this study provided important insights into the ergonomic risks associated with using wired and wireless IOSs during scanning. The increased muscle fatigue observed in some specific muscles during the fourth iteration of scanning simulations highlights the importance of taking adequate rest breaks during intraoral scanning procedures to minimize the risk of musculoskeletal disorders. The higher RMS EMG values and muscle fatigue observed with the wireless IOS may be attributed to the added weight of the wireless module and the need for frequent repositioning of the scanner owing to the limited range of motion. These results suggest that wireless IOSs may not be suitable for prolonged scanning procedures, especially in individuals already prone to musculoskeletal disorders. The analysis of the average EMG signal graphs in the present study reveals that, across all four consecutive tasks, higher EMG signals in the arm muscles (EDC and FDS) were observed when using the wireless IOS (Fig. 4). This suggests that the weight of the wireless IOS imposes a significant burden on the arm muscles from the first task onward. Furthermore, when examining shoulder muscles (SC and T), no differences were noted between the wired and wireless IOSs in the first three tasks. However, in the fourth task, particularly in the right SC muscle, a marked increase in EMG signals was observed with the wireless IOS. This indicates that while the initial repetitions did not significantly strain the shoulder muscles, by the fourth task, a direct load on these muscles became evident. Notably, with the wired IOS, a significant increase in EMG signals was observed in the left neck muscle (SCM) during the fourth repetition. This outcome could be attributed to the fact that, while the wireless IOS allowed the scanning process to be displayed on a monitor connected to a dental unit chair system, the wired IOS, limited by its cord, displayed the scanning process on the laptop's monitor (Figs. 2 and 3). Consequently, participants frequently had to rotate their necks towards the laptop to monitor the scanning process. These finding highlights that while repetitive intraoral scanning with the wired IOS may reduce the load on arm and shoulder muscles due to its lighter weight, the wireless IOS, unencumbered by a cord, offers the advantage of repositioning the monitor to minimize neck strain.

This study provides important insights into the potential risks associated with the consecutive use of wireless IOSs and highlights the need for dentists to consider ergonomic factors to ensure musculoskeletal health. Previous studies have reinforced the importance of ergonomic factors, such as the design of stools and chairs, in reducing muscle activation and fatigue among dentists [35–39]. A previous study focused on reducing lower back muscle activation [35], whereas the present study evaluated muscle activation and fatigue in the arm, neck, and shoulder muscles. Another previous study investigated the influence of different stool types on muscle activity and lumbar posture [37], whereas the present study evaluated the effect of wired and wireless IOSs on muscle activation and fatigue in specific muscles. Similar to the present study, previous studies have highlighted the importance of ergonomic interventions to improve musculoskeletal health in dental practice [35–39].

The present study and previous studies aimed to investigate the impact of ergonomics on musculoskeletal health in dentists [7, 8, 14]. However, previous studies have focused on different aspects of ergonomics and musculoskeletal health. A previous study focused on the design of dental scaling instruments and how different handle shapes affected muscle load and pinch force during a simulated periodontal work task [7]. This study provides guidance for dentists and dental hygienists in selecting dental scaling instruments that reduce the risk of work-related upper-extremity musculoskeletal disorders. Another study examined working postures during standard dental interventions and how these postures affect muscle activity and the risk of fatigue and injury [8]. This study suggests that combining sitting and standing postures can reduce the risk of fatigue and injury to certain muscles. A previous study evaluated the efficacy of different ergonomic supports (ergonomic stool, magnification lenses, and a combination of both) in reducing muscle activity of the neck and shoulder muscles during three posterior composite restoration procedures [14]. This study found that using ergonomic supports can effectively decrease muscle activity, with the combination of both supports providing the greatest decrease. The present study focused specifically on comparing muscle activation and fatigue between wired and wireless IOSs during consecutive intraoral scanning and found that the consecutive use of wireless IOSs may increase the risk of activation and fatigue in certain muscles, which may have clinical implications for dentists regarding ergonomics and musculoskeletal health.

In the present study, wireless IOS showed higher muscle contraction and fatigue than by wired IOS. In a previous study, muscle contraction and fatigue during the scanning process of IOS were compared with those during the treatment process using a handpiece for tooth preparation, and IOS showed higher muscle contraction and fatigue [6]. This was attributed to the weight of the medical device used. The IOS used in the previous study was considered heavy to work with for long periods owing to its weight of 280 g, which is heavier than the handpiece weighing approximately 100 g [6]. In the present study, the same company’s IOS (i700; MEDIT) was used, but there was a weight difference of 48 g owing to the addition of a wireless module and battery component in the wireless IOS (328 g for wireless and 280 g for wired). Therefore, it was demonstrated that a significant increase in muscle fatigue occurred during the fourth scanning process owing to this weight difference. According to previous studies, the weight of IOS varies from 113 to 585 g [5, 40], which means dentists need to pay special attention to the consecutive use of IOS in clinical practice. The selection criteria for IOS in the present study were predicated on the availability of both wired and wireless models from a single product line by a prominent manufacturer, chosen to specifically assess the impact of the wireless module's additional weight in isolation from other variables. Despite this, the substantial weight differences among IOS from various manufacturers suggest a need for additional clinical research involving a broader spectrum of products.

This study presents several limitations that warrant acknowledgment. The limitation is the small sample size, which may restrict the generalizability of the findings. Moreover, the present study excluded left-handed dentists and individuals with a history of musculoskeletal disorders, further limiting the scope of the results. Future research involving larger sample sizes and incorporating these participants could yield a more comprehensive understanding of the ergonomic risks associated with using wired and wireless IOSs. Another limitation is that this study did not explore the effectiveness of ergonomic interventions, such as adjustments in posture and instrument design, for reducing the risk of musculoskeletal disorders related to intraoral scanning procedures. Consequently, future studies should examine the effectiveness of these interventions in mitigating ergonomic risks associated with intraoral scanning procedures. The present study solely investigated muscle activity without measuring subjective symptoms of musculoskeletal discomfort or pain. Therefore, subsequent research should also assess subjective symptoms of discomfort or pain to better comprehend the ergonomic risks connected to intraoral scanning procedures. The clinicians participating in this study were relatively young and had limited clinical experience, potentially limiting the study's representation across a wider age range. Future research should thus aim to include a broader spectrum of ages to enhance the relevance of the findings. The study also did not consider the visibility of a clock to the dental practitioners during tasks, which could have influenced outcomes based on the participants' orientation relative to the timepiece. Additionally, individual differences in tool grip strength were not accounted for. Given the variability in grip strength among individuals, this factor should be considered in future studies. The study also did not differentiate between various types of wired and wireless IOSs, which have different weights and usability characteristics. Therefore, future experiments should involve a more diverse range of IOS models to provide a more nuanced understanding of their ergonomic impacts. Environmental or situational factors were not considered in this research, conducted in a standardized simulated environment. However, different environmental conditions could potentially influence the results, leading to potential confounding factors in the outcomes. In light of these considerations, future research should address these limitations and expand the scope of inquiry to provide deeper insights into the ergonomic risks and potential interventions associated with intraoral scanning procedures.

## Conclusion

Based on the findings of this preliminary clinical study, the following conclusions can be drawn:

1. The present study determined that wireless IOSs were associated with increased muscle activation and fatigue in specific muscles, such as those in the arm, neck, and shoulder, compared to wired IOSs.
2. Since no significant difference in work time (learning effect) was observed up to the fourth consecutive use of wired and wireless IOSs, the difference in EMG results can be attributed to the weight discrepancy between the two IOS types.
3. The wireless IOS presents a notable advantage by virtue of its wire-free design, facilitating the repositioning of the display monitor to a site that alleviates neck muscle strain.
4. The heightened muscle fatigue observed in certain muscles underscores the importance of incorporating sufficient rest breaks during intraoral scanning procedures to minimize the risk of musculoskeletal disorders.
5. The results imply that dentists should be aware of the potential risks linked to the consecutive use of wireless IOSs and implement measures to mitigate the impact of intraoral scanning on musculoskeletal health.

## Data Availability

The data sets used or analyzed during the current study are available from the corresponding author on reasonable request.
